# Supplementary material for: Acute kidney injury during an ultra-distance race
Source: PLoS One. 2019 Sep 25;14(9):e0222544. doi: 10.1371/journal.pone.0222544 (PMC6760777; doi:10.1371/journal.pone.0222544)
Supplement: Attachment — Submitted filename: Response to Reviewers.docx [file pone.0222544.s001.docx]

Dear Editor,

We thank the reviewers for their work and their interesting remarks.

Please consider the revised version of our manuscript.

Please find below the answers to the reviewers’ comments.

All substantial modifications appear in red and bold in the revised manuscript.

Sincerely yours.

Romain Jouffroy

Reviewer's Responses to Questions
**Comments to the Author**
Reviewer #1: The authors report on the temporal changes in kidney injury biomarkers before, during, and after an 80km ultra-distance race. The study was designed and conducted appropriately. The manuscript has potential and can benefit from some improved clarity with the results and associated conclusions. Additionally, the focus of the introduction and discussion can be expanded. A large focus of the introduction and discussion involves the AKI to CKD transition. Although this is a good point to make, the study had short term follow-up. The authors should expand these sections to include other exercise related AKIs, such as from “regular” marathons and spin classes. Several studies and numerous case reports have been published within the last few years, notably, one published in PLoS ONE. Additionally, Mansour et al. AJKD 2017, is an impactful study and should be discussed. The authors should add a discussion on the strengths and limitations of KIM-1 and NGAL, including the differences between blood and urine measurement.

**Answer: We thank the reviewer for the remark. In the revised version of the manuscript, the introduction and discussion sections were expended. Nevertheless, we believe that long-distance running is different from regular marathon running because the speed is quite lower, and most of races take place on trails with denivelation contrary to regular marathon on road. Another originality of our study is the kinetic of AKI biomarkers contrary to previous studies with different methodology. In previous studies, the impact of running is evaluated comparing before and after running biomarkers values.**

Also, the novelty the authors make regarding their study of an ultra-distance race rather than a “regular” marathon is probably not as clinically significant as the authors make it out to be. This should be adjusted throughout the manuscript including the last two paragraphs of the discussion.

**Answer: We thank the reviewer for this interesting suggestion. In the revised version of the manuscript, manuscript and the last two paragraphs of the discussion were adjusted in order to take into account the suggestion.**

Minor comments:
The in-text tables and figure legends (pages 8-10) are confusing to follow and should be moved to the proper section according to the journal’s author instructions. Also, the landscape formatting on the later pages needs to be fixed.

**Answer: We agree with this remark. In the revised version of the text, the landscape formatting was modified in order to accord with journal’s author instructions.**

*Introduction*
Page 4, line 3 – clarify what the 480,000 runners refers to specifically. Did 480,000 runners complete a marathon in that one year or in 2001, have 480,000 runners completed a marathon at some point in their life?

**Answer: We agree with this remark. In the revised version, the sentence was modified in order to avoid confusion.**

*Methods*
Page 5, line 8 – clarify what medical disease refers to. Is this specifically referring to chronic diseases? What about occasional headache or infections? Only systemic illness?

**Answer: We fully agree with this comment. The medical disease refers to chronic diseases.**

**A sentence has been added in the new version of the manuscript to clarify this point.**

Page 5, lines 9-10 – The sentence needs to be rewritten. I believe the authors intent to say that the participants had free access to food and water during and after the race. “ad libitum”.

**Answer: We thank the reviewer for this remark. In the revised version of the manuscript, the sentence was rewritten.**

The study protocol was approved in 2014, was this the same year the race occurred?

The authors should mention the year of the race. At least they mentioned the weather that day.

**Answer: We thank the reviewer for the remark and the suggestion. In the revised version of the manuscript, the year of the race and the day race weather were added.**

*Results*
Page 8 line 12 – Clarify the sentence to specify mean time of the race is referring to “mean race completion time”
**Answer: We thank the reviewer for this interesting suggestion. In the revised version of the manuscript, the sentence was rewritten.**

Table 1 – what is “Trial experience”?
**Answer: We thank the reviewer for this remark. In the revised version of the text, the term was changed for “seniority in running practice”.**

Page 10, line 18 – elevated urine creatinine does not necessarily reflect a “high level of urine concentration”, which is determined from the measurement of specific gravity or osmolarity. The high urine creatinine may simply be reflective of the higher blood creatinine level. Measurement of urinary osmolarity and electrolytes in stored samples would provide additional insight into kidney function during and after the race.

**Answer: We fully agree with this remark and thank the reviewer. In the revised version of the manuscript, the sentence was deleted in the results section and a sentence added in the discussion.**

How much of the rise in serum creatinine during the race is really a true reduction in CrCl vs. and increase in muscle release of creatinine? The authors should comment on this.
**Answer: We thank the reviewer for this very interesting remark. A sentence was inserted in the discussion to emphasize this very important point.**

It would be interesting to see the breakdown of the kidney injury biomarkers by KDIGO AKI stage. Were the biomarkers more elevated in these participants?

**Answer: We thank the reviewer for this interesting suggestion. A sentence was added in the results section to describe the kinetics of kidney injury biomarkers by KDIGO AKI stage.**

*Discussion*
Page 14, line 4 – Although the authors comment on AKI to CKD transition in other sections, they should specify here safe for “short term” kidney function as their study followed patients up to 9 days after the race.
**Answer: We fully agree with this suggestion and thank the reviewer. In the revised version of the manuscript, the sentence was modified in the abstract and manuscript conclusions.**

Page 14, lines 6-7 – This sentence is not accurate, the impact of long-distance running on kidney function has been studied. As mentioned earlier, the Mansour AJKD paper, and others.

**Answer: We thank the reviewer for this remark. The sentence was rewritten in order to underline the originality of our study showing the kinetic of kidney injury biomarkers.**

Since the authors talk about acute dehydration leading to ATN in the discussion, they should mention that the clinical parameters in table 2 do reflect any clinical evidence of dehydration and supports their finding of no major kidney injury.
**Answer: We thank the reviewer for this suggestion. A sentence was added in the results section to describe the kinetics of kidney injury biomarkers by KDIGO AKI stage.**

Page 15, line 7 – remove the word “the”

**Answer: We thank the reviewer for this remark. In the revised version of the manuscript, the term “the” was deleted.**

Page 15, line 8 – maybe hyaline and waxy casts are nonspecific but muddy brown urinary casts are not nonspecific and are indicative of tubular injury.

**Answer: We agree and thank the reviewer for this remark. The sentence was rewritten in order to be more accurate.**

Page 15, lines 14-15 – the assumption that urine was highly diluted before the race is incorrect. There is no evidence to support this claim. Additionally, what does “huge fluid consumption” actually mean?

**Answer: We agree with this remark and thank the reviewer. In the revised version of the manuscript, the sentence was deleted. By “huge fluid consumption”, we wanted to say that a possible explanation was the increase fluid intake before the race by most of runners.**

Reviewer #2: The Authors in their interesting work show that transient increase in creatinine is not accompanied to parallel increase of two urinary biomarkers of acute kidney injury (uNGAL/uCr and uKIM 1/uCr) (ultra-distance race) in trained athletes during intense physical effort.
**We thank the reviewer for his comment.**

Major criticisms
-The Authors show that uNGAL/uCr significantly decreases from baseline to day 9 after the physical effort, however in the discussion they state that “…all biomarker variation fully disappeared on day 9...” and “…urine levels of NGAL were similar at 80 km and at baseline after correction for urine dilution status”.

**Answer: We thank the reviewer for the remark. Figure 2B shows no difference between uNGAL/uCr between baseline (km 0) and day 9 whereas a significant difference was observed between baseline and the end of the race (km 80). The sentence, in the discussion, was rewritten in order to avoid confusion; we fully agree that “similar” and “not different” are not, statistically, synonymous.**

I believe that the decrease of uNGAL/uCr during the physical effort and at day 9 after the race deserves an appropriate discussion. Do the Authors have any data regarding the physical activity/diet in the short term before (ideally 7-10 days) and after the race?

**Answer: We thank the reviewer and fully agree with this comment.**

**We don’t have any data regarding the physical activity/diet in the short term before and after the race. An add was inserted in the revised version of the manuscript to emphasize the important impact of this limitation.**

Furthermore, it would be interesting to compare baseline athletes’ uNGAL/uCr with healthy control population.

**Answer: We thank the reviewer for this very interesting suggestion. Unfortunately, we don’t have data to compare baseline athletes’ uNGAL/uCr and healthy control population. A limitation was inserted in the revised version of the manuscript to underline this point.**

Finally it has been previously reported that uNGAL/uCr increases in athletes after 60 kg ultramarathon (Clin Chem Lab Med. 2012 Feb 14;50(9):1585-9. doi: 10.1515/cclm-2011-0954), please discuss (different population/analysis method?).
**Answer: We thank the reviewer for this reference. The reference has been added and potential explanations for the differences in studies results inserted in the discussion.**

Minor criticism
- eGFR should be reported in Table 2

**Answer: We thank the reviewer for this suggestion. In the revised version of the manuscript, eGFR (mean +/- SD) was added in Table 2.**

- I would suggest to report the number of subjects with blood test in Table 2 legend (as done by the Authors in Table 3 legend)

**Answer: We thank the reviewer for this suggestion. In the revised version of the manuscript, the number of subjects with blood test was added in Table 2 legend.**
